# Systems biology analysis of gene expression data and gene network reverse-engineering approaches reveal NFAT5 as a candidate biomarker in Inflammatory Breast Cancer

**DOI:** 10.1186/2051-1426-3-S1-P6

**Published:** 2015-08-14

**Authors:** Andrea Remo, Ines Simeone, Massimo Pancione, Pietro Parcesepe, Pascal Finetti, Halima Bensmail, Luigi Cerulo, Vittorio Colantuoni, Daniel Birnbaum, Franco Bonetti, Francois Bertucci, Erminia Manfrin, Michele Ceccarelli

**Affiliations:** 1 Department of Pathology, Mater Salutis Hospital, Legnago, Italy; 2 Qatar Computing Research Institute (QCRI), Qatar Foundation, Doha, Qatar; 3 Department of Science and Technology, University of Sannio, Benevento, Italy; 4 Department of Pathology and Diagnosis, University of Verona, Verona, Italy; 5 Department of Molecular Oncology, Institut Paoli-Calmettes, U1068 Inserm, Marseille, France

## 

Inflammatory Breast Cancer (IBC) is the most aggressive and highly metastatic form of breast cancer [1-3]. In a recent study [4], we analysed breast cancer with peritumoral neoplastic lymphovascular invasion (ePVI) in comparison with inflammatory breast cancer, showing that ePVI breast cancer have more clinicopathologic affinity than differences with the most aggressive cancer in the breast. Here, we aim to identify potential master regulators (MRs) that drive the expression pattern in IBC.

Transcriptomic (i.e., mRNA) data from 197 breast tumours were used for this analysis (GEO GSE23720) [5]. All tumours were classified as “IBC” (n=63) or “nIBC” (n=134). To identify novel MRs that drive the IBC phenotype, all expression data were analysed using a network-based strategy (ARACNe [6]) and Master Regulator Analysis (MRA)[7]. We chose to perform in-vivo IHC analysis, in two independent cohorts of IBCs (n = 39), nIBCs (n = 82) and normal breast tissues (n = 15), for the top significant Master Regulators: MGA, CTNNB1 and NFAT5. Biological validation confirmed that NFAT5 expression was higher in IBC than in nIBC (70% vs. 20%) and that the majority of NFAT5-positive IBC samples displayed NFAT5 nuclear expression in comparison with nIBC samples (89% vs. 12%).

We provide evidence that NFAT5 transcription factor could constitute a novel IBC biomarker that could help to identify the most aggressive forms of BC into routine clinical practice.
